# Study on improving collection feeding safety and quality of colostrum for very (extremely) low birth weight infants

**DOI:** 10.3389/fped.2022.1069719

**Published:** 2023-02-13

**Authors:** Hua Wang, Qiu-Fang Li, Xin-Fen Xu, Xiao-Li Hu

**Affiliations:** ^1^ Department of NICU, Women's Hospital, Zhejiang University School of Medicine, Hangzhou, China; ^2^ Department of Nursing, Women's Hospital, Zhejiang University School of Medicine, Hangzhou, China; ^3^ Department of Obstetrics, Women's Hospital, Zhejiang University School of Medicine, Hangzhou, China

**Keywords:** breastfeeding, colostrum, extremely low birth weight infants, oral administration, process optimization, very low birth weight infants

## Abstract

**Objective:**

This study aims to explore the difficulties related to fresh colostrum feeding for very (extremely) low birth weight infants (VLBWI/ELBWI) and optimize the colostrum administration process.

**Methods:**

The VLBWI/ELBWI who were admitted in the neonatal intensive care unit from January to December 2021, were enrolled as the experimental group, and an optimized colostrum feeding process was adopted. The VLBWI/ELBWI admitted from January to December 2020 were enrolled as the control group, and a conventional feeding process was adopted. The general situation of colostrum supply, number of adverse feeding events, maternal breastfeeding rate at the critical time points.

**Results:**

There were no significant differences between the baseline charatcteristics of the 2 groups. In the experimental group, compared with the control group, the time to first colostrum collection was significantly shorter (64.8% vs. 57.8% *p* < 0.05), and the rates of colostrum feeding (44.1% vs. 70.5% *p* < 0.001), and of maternal breastfeeding at 2 weeks after birth (56.1% vs. 46.7%, *p* < 0.05) and on the day of discharge (46.2% vs. 37.8%, *p* < 0.05) were significantly higher. Before and after process optimization, the average total time required for the time for nurses to receive the colostrum in the NICU reduced from 7.5 min/time to 2 min/time, and no feeding-related adverse events occurred.

**Conclusion:**

Fresh colostrum feeding process optimization for VLBWI/ELBWI, improves the colostrum feeding rate, shortens the time to first colostrum collection, saves the working time of nurses, and improves the maternal breastfeeding rate at key time points.

## 1. Introduction

It is reported that the prevalence of VLBW/ELBW premature infants in China is as high as 0.18% (1). The survival rate of such infants has been improved year by year (2), however, the incidence of hospital complications and mortality are still high (3).

Proper nutrition is the key factor to improving the survival rate and prognosis of VLBWI/ELBWI (4–6). Colostrum is the breast milk produced by the mammary glands within 5 days of child birth and is rich in immunoprotective agents (7). It protects infants from the risk of late onset sepsis, and other complications related to premature delivery (2). Therefore, colostrum is regarded as “liquid gold” (8), is as important as drugs and provides great protection to VLBWI/ELBWI (9). Furthermore, mothers of VLBWI/ELBWI face unique obstacles in expressing breast milk, such as maternal and infant health complications, stress related to the NICU environment, delay in normal physiological adaptation, separation of mother and infant, and delay in lactation, which may hinder them from successfully starting and maintaining breastfeeding (8). The current approach is that in addition to early micro tube feeding, medical institutions worldwide have begun to administer infants with colostrum orally. Oropharyngeal administration has become a special approach of colostrum feeding, and is suitable for VLBWI/ELBWI who cannot be fed orally or whose clinical conditions are unstable and has been included in many NICU standard practices (10). More and more studies have explored the impact of this method on the clinical outcome, feeding indicators, immune indicators, types of bacteria in the oropharynx, and detection rate of microbiota of premature infants (11). In these studies, the time, frequency, and methods of intervention were slightly different, but the details of how colostrum was collected and feeding safety promoted are not described in detail. Therefore, in this study, we optimized the nursing process of VLBWI/ELBWI colostrum feeding with relevant strategies and strengthened the quality control of colostrum collection, receipt by the NICU, feeding, collection equipment disinfection, and other related aspects in the ward. Details are as follows.

## 2. Study participants

The method used for sampling was a convenience sample. A total of 1,300 VLBWI/ELBWI hospitalized in the Neonatology Department of a Class III Grade A special hospital in Zhejiang Province from January 1, 2020 to December 31, 2021 were enrolled as the research participants. A total of 447 cases were excluded because the mothers had feeding contraindications, the premature infants were suspected or confirmed of suffering from genetic metabolic diseases (such as galactosemia), family members gave up treatment, or the premature infants were transferred to other hospitals. Finally, 853 cases were included in this study. Of these, 435 cases that were admitted from January 1 to December 31, 2021, were assigned to the experimental group, and 418 cases that were admitted from January 1 to December 31, 2020, were assigned to the control group. Parents/guardians of all participants provided signed informed consent. This study was approved by the hospital ethics committee, ethical batch No: IRB-20220045-R.

## 3. Study methods

### 3.1. Intervention methods in the experimental group

#### 3.1.1. Formulation of nursing process for colostrum oral administration

This study was carried out during the COVID-19 pandemic. Drawing lessons from the suggestions of many expert groups worldwide on improving the quality of early colostrum feeding and evidence-based practice (10, 12–15), the nursing process of colostrum feeding was optimized based on the actual situation of the hospital and included five aspects—training of medical personnel, collection of sticky colostrum, safe transportation of colostrum, standardized detoxification of pumping accessories, and electronic data processing.

#### 3.1.2. Implementation of optimized nursing process

**Figure 1:**
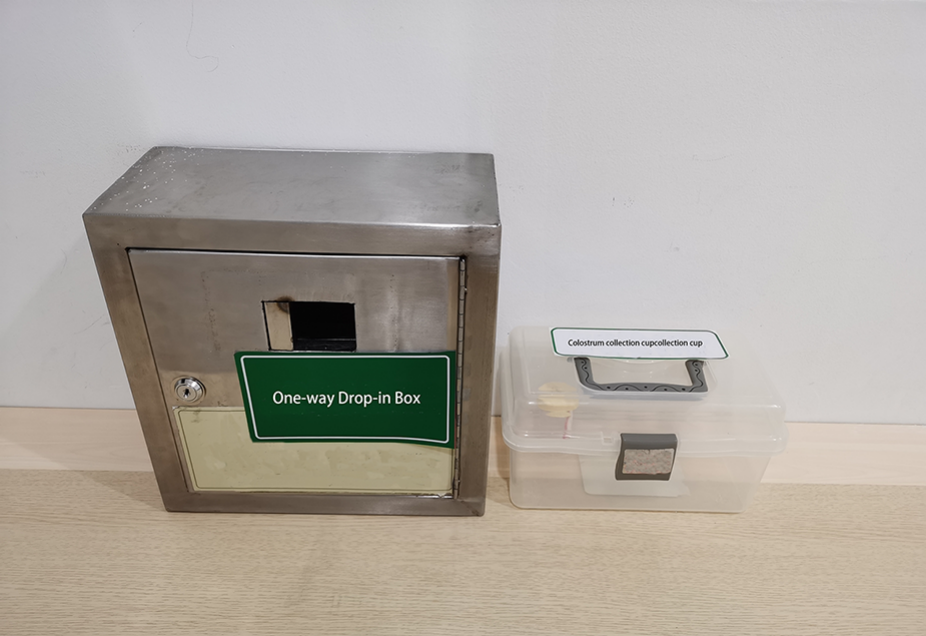
Colostrum collection cup and one-way drop-in box.

**Figure 2:**
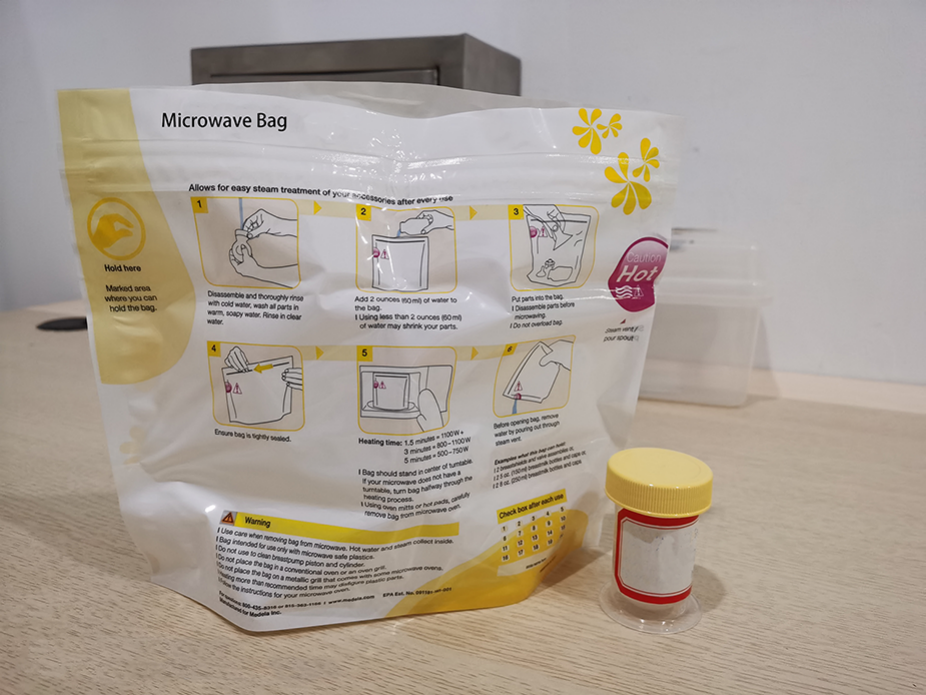
Quick clean bag.

(1) Staff education: The training module was established in the NICU. NICU medical staff, breastfeeding consultants, and new staff in the department received refresher training. The training included:
(i) Education on the importance of colostrum feeding: A standard breastfeeding consultation module was established—“Maximizing colostrum production by newly lactating mothers via hand expression and the use of breast pumps – education and training strategy.” The education module is based on A Training Compendium on Providing Mothers' Own Milk in NICU Settings(exclusively published on www.LactaHub.org on 4 August 2020) and consists of two parts. The first part is a slide presentation introducing the important benefits of colostrum for premature infants. Fresh breast milk refers to breast milk that is directly fed to the infant without freezing or heating, within 4 h after expression, with immune cells and stem cells preserved.The second part is composed of video clips that combine hand expression and breast massage with simultaneous breast pumping to maximize the production of breast milk. The trained breastfeeding consultant made routine morning rounds every day and entered the maternity ward' to provided education and guidance on the importance of colostrum secretion and feeding, to new mothers and families.
(ii) In the experimental group, two breastfeeding consultants certified by the Provincial Maternal and Child Health Association with more than 10 years of working experience in the NICU were assigned.Breastfeeding consultants and NICU nurses were trained on early breast milk expression techniques and learned colostrum “posture drainage” techniques. The breastfeeding consultants were required to teach the techniques at the bedside when the newly lactating mothers and their family members conducted hand expression/breast pumping and syringe-based colostrum collection for the first time.
(iii) Education on effective perinatal infant protection strategy targeting the novel coronavirus Covid-19 was carried out, emphasizing that mothers should wear masks, maintain good hand hygiene, and avoid coughing or sneezing on breastfeeding accessories, breast milk storage containers, and their breasts (16).
(2) Collection of sticky colostrum: The newly lactating mother was encouraged to stand upright or lie on the side during colostrum collection, allowing the colostrum with low viscosity to flow through the breast pump through “body posture drainage.” If the newly lactating mother could not stand or lie on her side, she could lie in the supine position and colostrum was collected *via* a syringe with the help of a breastfeeding consultant or family member. In the experimental group, family members were allowed to participate in the subsequent collection process, contributing to ensure the continuity of colostrum feeding. The steps for using the breast pump and hand expression to collect colostrum were included in the education and nursing training in the NICU and were strengthened by breastfeeding consultants.
(3) Safe transportation of colostrum: Based on the feeding schedule of NICU infants, the family members collected colostrum at the feeding time points (3 h apart) 1 h in advance, which was transported to the NICU immediately and fed eight times to the respective infant, to ensure that the fresh colostrum was used within 1 h. The NICU admission disposal room was equipped with a one-way delivery box (see Figure 1), that is, the colostrum collection cup could not be taken out after being placed inside, to ensure safe delivery without the need for on-duty personnel. After the NICU nurse unlocked the delivery box and collected the cups at the feeding time point, the empty box was locked, and the collection cups after the last use were uniformly placed in a plastic frame. The delivery box is made of stainless steel. NICU nurses disinfect and wipe it at least once a day and use alcohol pads to wipe the body of the collection cup. Notes were attached to the delivery box reminding family members to tighten the cap of the collection cup, make sure the labels on the bottle body remained intact, and to take back the contaminated collection cup for cleaning and disinfection. Fresh breast milk can be stored at room temperature for 3 h (17), hence cold chain facility is not required for the one-way delivery boxes.
(4) Standard sterilization of breast pump accessories and collection cups: The microwave oven can kill 99.99%–100% of bacterial propagules such as Escherichia coli and Staphylococcus aureus within 3 min after being exposed to the lowest output power (540 W) (18). It features rapid temperature rise, strong bactericidal power, and short disinfection time, and is suitable for disinfection and sterilization of small pieces or articles with small thickness. In addition, the contents of microwave disinfection must contain water (19). Therefore, it is better to disinfect accessories and collection cups that are wet or to place them in containers with appropriate amount of water before microwave sterilization. We chose special disinfection bags for microwave ovens (see Figure 2). The bag was filled with no less than 60 ml of drinkable water, sealed, and microwaved for 3 min at a high setting (800–1100 W) in a microwave oven. A disinfection bag can be reused up to 20 times, and the bags were marked after each use.
(5) Electronic data processing: We created an electronic real-time record format using Tencent documents; the access link was shared with the clinical teams. The breastfeeding consultant updated the form every morning and listed the VLBWI/ELBWI admitted to the neonatal department within 24 h of their admission. The breastfeeding consultants entered details on breast stimulation, colostrum collection and feeding, milk secretion, and other data in real time using smartphones. The breastfeeding consultant conducted appropriate data maintenance in the subsequent week.

### 3.2. Oral administration of colostrum to the control group

Routine oral administration was adopted for the control group (20). A written colostrum feeding protocol was developed in the NICU. Posters encouraging colostrum feeding were posted in a prominent position in the neonatal ward. A brochure on the importance of breastfeeding was provided on the WeChat platform. NICU staff encouraged the newly lactating mothers to express milk every 2–3 h. The hospital provided hospital-level breast pumps and milking rooms. NICU nurses provided bedside education and guidance. Colostrum was stored in milk storage bags or disposable aseptic cups, transported by family members, and stored in the refrigerator of the ward after being received by nurses. The colostrum was heated in a water bath before feeding. NICU nurses received training on how to perform oropharyngeal administration of colostrum and recorded the details of colostrum feeding/oropharyngeal administration.

### 3.3. Evaluation indexes and data collection methods

#### 3.3.1. General condition of colostrum supply

(1) The colostrum feeding rates of the two groups were collected, recorded, and compared by breastfeeding consultants.
(2) The time to first colostrum collection was compared between the two groups, and related data were automatically acquired from Tencent documents and summarized.
(3) The average total time for receiving the colostrum at the NICU for each feeding time point between the two groups was collected, recorded, and compared by the NICU nurses uniformly.

#### 3.3.2. Safety assessment indexes

Adverse events related to colostrum feeding, including intestinal infection caused by colostrum contamination (21, 22), feeding errors, or omissions, were collected and recorded by NICU nurses. Nosocomial intestinal infections could be due to bacterial infection in the gastrointestinal tract after ingesting contaminated colostrum due to improper colostrum collection or transportation or improper disinfection of breast pump accessories and collection cups. Diagnosis of Nosocomial infections was clinically based as per criteria described by Shao et al. (23).

#### 3.3.3. Maternal breastfeeding rate

(1) The maternal breastfeeding rates two weeks after birth between the two groups were collected, recorded, and compared by breastfeeding consultants.
(2) The maternal breastfeeding rates on the day of discharge between the two groups were collected, recorded, and compared by breastfeeding consultants.

### 3.4. Statistical methods

Data were analyzed using statistical software SPSS26.0. Normally distributed measurement data were expressed as mean ± standard deviation $\left(\bar{X}\pm \mathrm{SD}\right)$ and compared between the two groups using independent samples *t*-test. Non-normally distributed measurement data were expressed as median and interquartile range, and compared using nonparametric test. Count data were expressed as number of cases, proportion, or percentage (%), and compared between the two groups using Chi-square test. *P* < 0.05 was considered statistically significant.

## 4. Results

### 4.1. Comparison the characteristics of infants between the two groups

There were no significant differences in the characteristics of infants between the two groups (See Table 1).

**Table 1:** Comparison of infants in both groups.

Group	Gestational age (week, median)	Birth weight (g, median)	Small-for-gestational age infants (%)
Control	30	1272.50	20
Experimental	29.86	1220.00	31
	*U = *85,889.50	*U = *84,182.00	*χ*^2 ^= 2.33

### 4.2. Comparison of overall condition of colostrum supply between the two groups

Before and after adoption of the optimized process, the colostrum feeding rate increased significantly from 44.1% to 70.5% (*p* < 0.001), and the median time to first colostrum collection of the two groups decreased significantly from 64.8 h to 57.8 h (*p* < 0.05). The average total time required by nurses to receive colostrum at each feeding time point decreased from 7.5 min/time to 2 min/time (See Table 2).

**Table 2:** Comparison of colostrum supply.

	Colostrum feeding rate%	Time to first colostrum acquisition (hour)	Average total time required by nurses to receive colostrum at each feeding time point (minutes/time)
Control group	44.1	64.8	7.5
Experimental group	70.5	57.8	2
	*χ*^2 ^= 61.537, *P *< 0.001	*Z *= 2.384, *P *< 0.05	–

### 4.3. Comparison of colostrum feeding-related adverse events

Adverse events related to colostrum feeding omission decreased from 4 cases before adoption of the optimized process to 0 cases after adoption, and there was no colostrum feeding related intestinal infection.

### 4.4. Maternal breastfeeding rate

In the experimental group, compared with control group, the maternal breastfeeding rate at 2 weeks after birth was significantly higher (56.1% vs. 46.7%, *p* < 0.05), including on the day of discharge (46.2% vs. 37.8%, *p* < 0.05). (see Table 3).

**Table 3:** Comparison of maternal breastfeeding rates at key time points.

	Maternal breastfeeding rate 2 weeks after birth%	Maternal breastfeeding rate on the day of discharge%
Control group	46.7	37.8
Experimental group	56.1	46.2
	*χ*^2 ^= 7.607, *P *< 0.05	*χ*^2 ^= 6.182, *P *< 0.05

## 5. Discussion

### 5.1. Optimizing the colostrum administration nursing process can improve colostrum feeding rate and shorten the time to first colostrum collection

Colostrum milking and collection need to be learned as a special skill. The results of this study revealed that in the control group, NICU nurses reported that it was difficult to collect colostrum (it was quite time-consuming), involved heavy nursing tasks, and management often replaced detailed lactating consultation. It was decided that the breastfeeding consultant should assist in colostrum expression and then pass the skill on to the newly lactating mothers and their family members. This method improved breastfeeding consultation and provided early education and evaluation for mothers and families of small premature infants in a timely manner and improved the compliance of newly lactating mothers.

Colostrum is highly viscous and has small volume. For some newly lactating mothers with premature infants, it is particularly difficult to collect 10–20 ml of breastmilk every day for 3 days after birth. Our team provided some specific techniques to help them collect colostrum. If a newly lactating mother could change her posture, we encouraged her to stand upright or lie on her side, as these postures help in collecting colostrum. If they were unable to do so during breast pumping due to postoperative pain, acute or chronic diseases, and delivery complications, then they needed to remain in the supine position, which is an obstacle to using the breast pump alone. Two breastfeeding consultants made daily ward rounds, screened the above high-risk groups, and then designated the use of syringes at the bedside to collect colostrum, which was then transferred to the colostrum collection cup and immediately sent to the NICU with the infants' relatives, after labeling. This intervention shortened the first colostrum feeding time. If the newly lactating mother was unable to collect colostrum by herself, temporarily with her permission, the breastfeeding consultant taught and demonstrated the correct techniques to their partners or important family members; after gaining sufficient proficiency, family members were allowed to participate in the subsequent collection process, thus ensuring the continuity of colostrum feeding.

The colostrum collection cup with a capacity of 35 ml can be directly connected to an electric breast pump (to avoid secondary contamination); the arc-shaped bottom design can ensure more micro colostrum is collected, ensuring there is no additional loss of milk hanging on the wall, and enhance the enthusiasm of lactating mothers to collect colostrum. The syringe used for colostrum collection is equivalent to the syringe for intravenous medication, which has certain potential safety hazards, therefore, colostrum must be transferred to a colostrum collection cup in the last step.

To improve communication between clinical team members, we established an electronic data collection form—Tencent document on the DingTalk information platform to ensure real-time and accurate data capture, and to ensure that people being trained and the NICU and medical staff could promptly evaluate the lactation situation and provide corresponding guidance.

This study revealed that the colostrum feeding rate increased from 44.1% to 70.5%, which was similar to the research results of Manerkar et al. (24), which saw an increase from 4.36% to 68.21%. Compared to international studies, the time to first colostrum collection of the experimental group in this study still has a large space for improvement. Research teams (25–27) reported that colostrum from their respective experimental groups was collected within 24 h after birth. The limitation of this study is that there was a lack of trained peer counselors, and the application rate of hospital-level breast pumps was low (8.5%), which provide a clinical basis for further process re-optimization. It is recommended that the NICU implement lactation ward rounds, increase the availability of hospital-level breast pumps or lease hospital-level electric breast pumps, provide colostrum collection kits, pump components, and hands-free pumping bras for free, and encourage newly lactating mothers to participate in oral administration of colostrum (28).

### 5.2. Optimizing the colostrum feeding nursing process can reduce adverse events of colostrum feeding and ensure the safety of colostrum feeding

Repeated use of colostrum collection cups and milk pumping/sucking accessories could easily lead to bacterial contamination and growth on the surface. Dong et al. (29) emphasized that training on how to clean breastfeeding equipment should be provided to newly lactating mothers. Considering the safety of electricity in the obstetric ward, family members cannot use boiling water-based sterilizers, moreover, during the COVID-19 pandemic, the closed management of the ward could not achieve home heating and boiling, therefore, in this study, the experimental group introduced microwave oven disinfection bags, which only required 3 min to disinfect the accessories. This approach was fast, effective, and convenient and no intestinal infection occurred caused by colostrum contamination during use. It is suitable for hospital use and is worthy of clinical popularization.

The results of this study revealed that although colostrum was collected and sent to the NICU in the control group, there was risk of feeding omission. This is because the family members randomly squeezed and sent the colostrum instead of referring to the feeding schedule of the NICU, while NICU nurses did not know that colostrum was placed in the feeding cart or stored in the refrigerator. Therefore, we decided that colostrum should be placed at a designated position and at a designated time by family members, and nurses would prioritize colostrum feeding after unified collection, to avoid missed feeding or misuse.

### 5.3. Optimizing the colostrum feeding nursing process can effectively save working hours and further improve work efficiency

The one-way delivery device can temporarily store colostrum for a short time, which is safe, reliable, efficient, and convenient. Utilizing it, family members can deliver colostrum by themselves 24 h a day without waiting, and nurses can collect colostrum in a centralized manner, reducing multiple handovers, signing, and unnecessary communication costs, and improve the work efficiency. During the COVID-19 pandemic, the frequency of direct contact significantly reduced to avoid cross-infection caused by the flow of people, thus disturbance to the work and life of both doctors and patients could be minimized, and the willingness of the two sides to use colostrum was further improved.

### 5.4. Optimizing the colostrum feeding nursing process can improve the maternal breastfeeding rate

The starting and establishment of lactation should be completed within 2 weeks after delivery (key window period for lactation), if the standard is not met, the subsequent milk volume will be seriously affected. The maintenance of lactation is usually measured by whether the child still receives maternal breastfeeding when discharged from the NICU (30). Heine et al. pointed out that (31), the possibility of maternal breastfeeding at discharge was minimum for VLBWI/ELBWI, which requires special support, and early colostrum and other interventions should be advocated. Therefore, 2 weeks after delivery and at discharge are the critical time points to judge whether the lactation is started and maintained successfully. Kalluri (32), Bagga (33) et al. revealed in their study that early implementation of maternal breastfeeding support in the NICU could improve the initiation of maternal breastfeeding for VLBWI/ELBWI, and the lactating mothers could successfully maintain maternal breastfeeding at discharge. Our study further proved that the attention and intervention measures of early colostrum collection could positively affect the duration of maternal breastfeeding and improve the maternal breastfeeding rate at critical time points.

## 6. Conclusion

Simple quality improvement measures such as training pf medical workers, providing lactation counseling to newly lactating mothers and their families, assisting them in collecting colostrum, and arranging the expression and delivery time according to the feeding time of the NICU can improve colostrum feeding rate. Most importantly, fresh colostrum should be delivered to the NICU as soon as possible and administered directly to VLBWI/ELBWI. By optimizing the process, we can realize colostrum tube feeding and oral administration, ensuring minimum waste and optimal utilization of colostrum.

## Data Availability

The original contributions presented in the study are included in the article/Supplementary Material, further inquiries can be directed to the corresponding author.

## References

[1] Charles E Hunt KA Harris C Hickey A Greenough A. Small for gestational age and extremely low birth weight infant outcomes. J Perinat Med. (2019) 47(2):247–51. 10.1515/jpm-2018-0295 30335614

[2] Wang B Sun J Sun Y Li N Li X Song X A clinical analysis of very and extremely low birth weight preterm infants. Am J Transl Res. (2021) 13(8):9395–403. PMID: ; PMCID: 34540058 PMC8430080

[3] Research Collaboration Group on Ultra Immature Infants and Ultra Low Birth Weight Infants. Short term outcomes and their related risk factors of extremely preterm and extremely low birth weight infants in guangdong province. Chin J Contemp Pediatr. (2019) 57(12):934–42. 10.3760/cma.j.issn.0578?1310.2019.12.008 31795560

[4] Hsiao CC Tsai ML Chen CC Lin HC. Early optimal nutrition improves neurodevelopmental outcomes for very preterm infants. Nutr Rev. (2014) 72(8):532–40. 10.1111/nure.12110 24938866

[5] McNelis K Fu TT Poindexter B. Nutrition for the extremely preterm infant. Clin Perinatol. (2017) 44(2):395–406. 10.1016/j.clp.2017.01.012 28477668

[6] Myrhaug HT Brurberg KG Hov L Markestad T. Survival and impairment of extremely premature infants:a meta-analysis. Pediatrics. (2019) 143(2):e20180933. 10.1542/peds.2018-0933 30705140

[7] Tanaka K Nakamura Y Terahara M Yanagi T Nakahara S Furukawa O Poor bifidobacterial colonization is associated with late provision of colostrum and improved with probiotic supplementation in low birth weight infants. Nutrients. (2019) 11(4):839. 10.3390/nu11040839 31013872 PMC6520773

[8] Obaid M Igawa T Maxwell A Murray YL Rahman A Aboudi D “Liquid gold” lactation bundle and breastfeeding rates in racially diverse mothers of extremely low-birth-weight infants. Breastfeed Med. (2021) 16(6):463–70. 10.1089/bfm.2020.0322 34042464

[9] Luna MS Martin SC Gómez-de-Orgaz CS. Human milk bank and personalized nutrition in the NICU: a narrative review. Eur J Pediatr. (2021) 180(5):1327–33. 10.1007/s00431-020-03887-y 33244710 PMC7691070

[10] Haase B Johnson TS Wagner CL. Facilitating colostrum collection by hospitalized women in the early postpartum period for infant trophic feeding and oral immune therapy. J Obstet Gynecol Neonatal Nurs. (2018) 47(5):654–60. 10.1016/j.jogn.2018.05.003 30196807

[11] Li YY Zhao X Li GZ Liu XQ Zhong XL Li MM The effects of oropharyngeal colostrum administration in preterm infants:a systematic review. Chin J Nursing. (2019) 54(5):753–9. 10.3761/j.issn.0254-1769.2019.05.027

[12] Meier PP Johnson TJ Patel AL Rossman B. Evidence based methods that promote human milk feeding of preterm infants: an expert review. Clin Perinatol. (2017) 44(1):1–22. 10.1016/j.clp.2016.11.005 28159199 PMC5328421

[13] Mercado K Vittner D McGrath J Rossman B. What is the impact of NICU-dedicated lactation consultants? An evidence-based practice brief. Adv Neonatal Care. (2019) 19(5):383–93. 10.1097/ANC.0000000000000602 30893096

[14] Deng YF He F Fu BL Guo XP He JY Liang QX Evidence-based practice to promote breast milk expression among postpartum women with neonatal-maternal separation. Chin J Nursing. (2020) 55(1):22–7. 10.3761/j.issn.0254-1769.2020.01.003

[15] Expert consensus core group on breast milk use in neonatal intensive care unit, Cao Y Li ZH Han SP Zhang QS Li LL Expert consensus on breast milk use in neonatal intensive care unit. Chin J Evidence Based Pediatr. (2021) 16(3):171–8. 10.3969/j.issn.1673-5501.2021.03.001

[16] Wei WL, Wu SF, Li ZW, Li HJ, Qu H, Yao CL

[17] Jiang M Luo BR. Clinical manual of breastfeeding. Beijing: People's Health Publishing House (2021). 194.

[18] Liu M Ma ZZ. Observation and comparison on two disinfection methods with medical wiping towel. Chin J Practical Nursing. (2011) 27(1):51–4. 10.3760/cma.j.issn.1672-7088.2011.01.025

[19] You PT Wang NH. Laboratory observation on the disinfection effect of microwave on tableware. Chin J Disinfection. (2006) 23(6):512. 10.3969/j.issn.1001-7658.2006.06.034

[20] Li QF Wang H Liu ZY Xu XF. The immune effects of mother colostrum daub by sublingual mucosal method in very low and extremely low birth weight infants. Chin J Nursing. (2018) 53(12):1424–8. 10.3761/j.issn.0254-1769.2018.12.003

[21] Huang J Lin XZ Zheng Z Zhao XY Gao JH Huang LH Short-term effects of mother's Own fresh milk feeding on very/extremely preterm infants. Chin J Neonatol. (2020) 35(3):169–74. 10.3760/cma.j.issn.2096-2932.2020.03.003

[22] Wu B Li QF Yi SY Lin R Shang GXJ Zhang R The construction and application of intelligent information management system for donor human milk bank. Chin J Nursing. (2021) 56(2):189–93. 10.3761/j.issn.0254-1769.2021.02.005

[23] Shao XM Ye HM Qiu XS. Practical neonatology. 5th ed Beijing: People's Medical Publishing House (2019). p. 622–3.

[24] Manerkar S Kalamdani P Patra S Kalathingal T Mondkar J. Improving early colostrum feeding in a tertiary neonatal intensive care unit: a quality improvement initiative. Breastfeed Med. (2022) 17(2):143–8. 10.1089/bfm.2021.0173 34726511

[25] Bashir T Reddy KV Kiran S Murki S Kulkarni D Dinesh P. Effect of colostrum given within the 12 h after birth on feeding outcome, morbidity and mortality in very low birth weight infants:a prospective cohort study. Sudan J Paediatr. (2019) 19(1):19–24. 10.24911/SJP.106-1540825552 31384084 PMC6589796

[26] Porta R Miralles N Paltrinieri A Ibáñez B Giménez J Roca T A breast milk pump at the bedside: a project to increase milk production in mothers of very low birth weight infants. Breastfeed Med. (2021) 16(4):309–12. 10.1089/bfm.2020.0122 33351698

[27] Fallahi M Shafiei SM Taleghani NT Shariati MK Noripour S Pajouhandeh F Administration of breast milk cell fractions to neonates with birthweight equal to or less than 1800 g: a randomized controlled trial. Int Breastfeed J. (2021) 16(1):63. 10.1186/s13006-021-00405-0 34425828 PMC8383348

[28] Digal KC Upadhyay J Singh P Shubham S Grover R Basu S. Oral care with Mother's Own milk in sick and preterm neonates: a quality improvement initiative. Indian J Pediatr. (2021) 88(1):50–7. 10.1007/s12098-020-03434-5 32638336

[29] Dong DD Ru XF Huang XF Sang T Li S Wang Y A prospective cohort study on lactation status and breastfeeding challenges in mothers giving birth to preterm infants. Int Breastfeed J. (2022) 17(1):6. 10.1186/s13006-021-00447-4 35012631 PMC8751123

[30] OuYang X Yang CY Xiu WL Hu YH Mei SS Lin Q. Oropharyngeal administration of colostrum for preventing necrotizing enterocolitis and late-onset sepsis in preterm infants with gestational age 32 weeks: a pilot single-center randomized controlled trial. Int Breastfeed J. (2021) 16(1):59. 10.1186/s13006-021-00408-x 34419090 PMC8379587

[31] Heine E Mehler K Schöpping M Ganesh L Klein R Kribs A Privacy, early colostrum, and gestational age are associated with exclusive breastfeeding in preterm and sick term infants. Z Geburtshilfe Neonatol. (2021) 225(4):346–52. 10.1055/a-1524-3328 34384133

[32] Kalluri NS Burnham LA Lopera AM Stickney DM Combs GL Levesque BM A quality improvement project to increase mother's Milk use in an inner-city NICU. Pediatr Qual Saf. (2019) 4(5):e204. 10.1097/pq9.0000000000000204 31745507 PMC6805104

[33] Bagga N Kurian S Mohamed A Reddy P Chirla DK. A quality initiative to improve mother's Own milk feeding in preterm neonates. Breastfeed Med. (2020) 15(10):616–21. 10.1089/bfm.2020.0033 32799551

